# Anti-CD321 antibody immunotherapy protects liver against ischemia and reperfusion-induced injury

**DOI:** 10.1038/s41598-021-85001-2

**Published:** 2021-03-18

**Authors:** Enzhi Yin, Takeshi Fukuhara, Kazuyoshi Takeda, Yuko Kojima, Kyoko Fukuhara, Kenichi Ikejima, Hisashi Bashuda, Jiro Kitaura, Hideo Yagita, Ko Okumura, Koichiro Uchida

**Affiliations:** 1 Atopy Research Center, Juntendo University Graduate School of Medicine, Tokyo, Japan; 2 Department of Neurology, Juntendo University Graduate School of Medicine, 2-1-1 Hongo, Bunkyo-ku, Tokyo, 113-8421 Japan; 3 Laboratory of Cell Biology, Research Support Center, Juntendo University Graduate School of Medicine, Tokyo, Japan; 4 Department of Biofunctional Microbiota, Juntendo University Graduate School of Medicine, Tokyo, Japan; 5 Laboratory of Morphology and Image Analysis, Research Support Center, Juntendo University Graduate School of Medicine, Tokyo, Japan; 6 Department of Gastroenterology, Juntendo University Graduate School of Medicine, Tokyo, Japan; 7 Department of Immunology, Juntendo University School of Medicine, Tokyo, Japan; 8 Juntendo Advanced Research Institute for Health Science, Juntendo University School of Medicine, 2-1-1 Hongo, Bunkyo-ku, Tokyo, 113-8421 Japan

**Keywords:** Immunology, Transplant immunology

## Abstract

The prognosis of the liver transplant patients was frequently deteriorated by ischemia and reperfusion injury (IRI) in the liver. Infiltration of inflammatory cells is reported to play critical roles in the pathogenesis of hepatic IRI. Although T lymphocytes, neutrophils and monocytes infiltrated into the liver underwent IRI, we found that neutrophil depletion significantly attenuated the injury and serum liver enzyme levels in a murine model. Interestingly, the expression of CD321/JAM-A/F11R, one of essential molecules for transmigration of circulating leukocytes into inflammatory tissues, was significantly augmented on hepatic sinusoid endothelium at 1 h after ischemia and maintained until 45 min after reperfusion. The intraportal administration of anti-CD321 monoclonal antibody (90G4) significantly inhibited the leukocytes infiltration after reperfusion and diminished the damage responses by hepatic IRI (serum liver enzymes, inflammatory cytokines and hepatocyte cell death). Taken together, presented results demonstrated that blockade of CD321 by 90G4 antibody significantly attenuated hepatic IRI accompanied with substantial inhibition of leukocytes infiltration, particularly inhibition of neutrophil infiltration in the early phase of reperfusion. Thus, our work offers a potent therapeutic target, CD321, for preventing liver IRI.

## Introduction

Ischemia and reperfusion injury (IRI) in the liver is an inevitable consequence of a number of common clinical situations and operative procedures that include circulatory shock, stroke, myocardial infarction and hepatectomy^1^. Especially in liver transplantation, IRI is recognized as the critical event to modulate prognosis of the patients^2^. Nevertheless, there is no practical and effective therapy to avoid hepatic IRI except for ischemic preconditioning^3^. Thus, it is important to reveal the mechanisms of hepatic IRI, particularly in reperfusion phase, for developing novel therapeutic strategies^4^.

It has been shown that the pathophysiological process of IRI is consisted with two phases, ischemia phase followed by reperfusion phase^5^. Ischemia potentiates oxidative-stress mediated tissue damage due to the reentry of oxygenated blood^6,7^. Reactive oxygen species (ROS) activate Kupffer cells in concomitant with producing inflammatory cytokines, chemokines and other mediators. Sequentially in reperfusion phase, an acute inflammatory response will be triggered by chemoattraction and activation of leukocytes, resulting in massive tissue damage^8,9^.

During these processes, it has to be evaluated whether trans-endothelial migration (TEM) of leukocytes is a key mechanism in prognosis as well as a therapeutic target in hepatic IRI. Inflammatory leukocytes undergo multiple and sequential steps under the strict control by coordinated surface molecules, then there are several reports showing the antibodies to block TEM^10^. Among the molecules on vascular surface, CD321 (a.k.a. Junctional Adhesion Molecule A or F11R) has been characterized as one of the essential molecules for TEM at the site of inflammation^10^. CD321 localizes at intercellular tight junction of endothelial cells to support barrier function under physiological conditions, but ischemia or inflammation stimulates subcellular relocalization as well as diapedesis of lymphocyte via LFA-1 binding^11,12^. Hepatic IRI was exaggerated in constitutive CD321-deficient mice despite reduction of neutrophil transmigration^13^. These results suggested hepatic IRI is regulated differently in multiple steps by CD321. Although the role of CD321 in the infiltration of neutrophil or homeostatic recovery of barrier function after hepatic IRI is unclear, it has been shown that inhibition of CD321 protein with anti-CD321 monoclonal antibody (mAb) (clone BV11) blocked neutrophil recruitment in experimental meningitis model^14^. Then, we here examined the therapeutic efficiency of anti-CD321 mAb (90G4) treatment in hepatic IRI model.

## Results

### Neutrophil depletion inhibited liver damage in a murine hepatic IRI model

**Figure 1:**
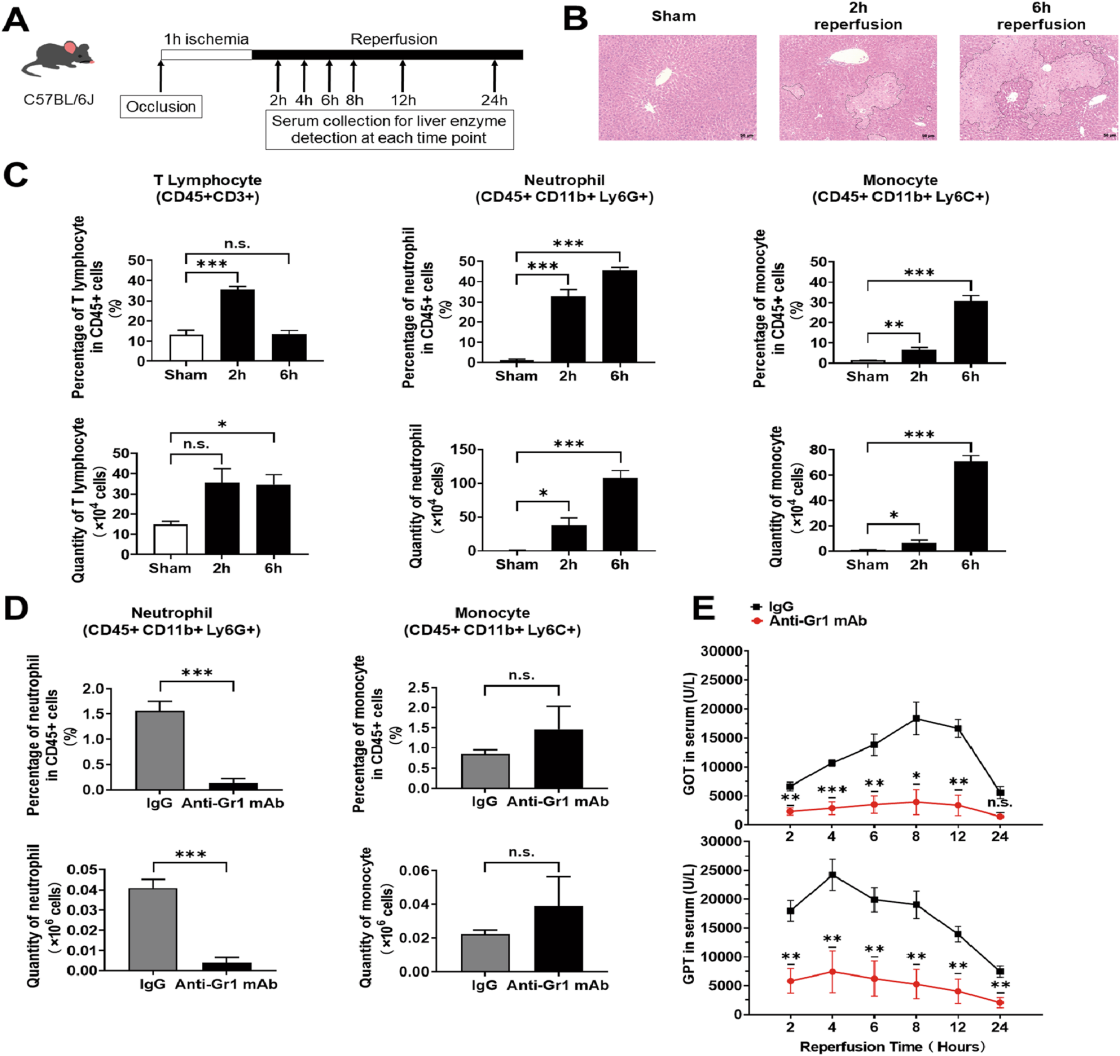
Hepatic IRI was alleviated by neutrophil depletion. (**A**) Protocol for a murine hepatic IRI model. (**B**) Representative images of hematoxylin and eosin staining of ischemic liver at indicated time after reperfusion. Necrosis areas are surrounded by dashed line. Image of the liver of the sham-operated mice were not distinguishable between 2 and 6 h after the sham-operation, thus the image of the liver at 6 h after sham-operation is demonstrated. (**C**) Percentage and the number of intrahepatic infiltrating T lymphocytes, neutrophil and monocyte in ischemic livers at 2 h and 6 h after reperfusion (n = 3–5 in each group). (**D**) Mice were treated with anti-Gr1 mAb at 6 and 4 days before the experiments, and PBMCs were obtained promptly before the experiments to confirm neutrophil depletion. Percentage and number of neutrophils and monocytes in PBMCs of anti-Gr1 mAb-treated and control (rat IgG2b)-treated mice are presented (n = 3 in each group). (**E**) Sera were collected at the indicated time after reperfusion from the hepatic IRI mice treated with anti-Gr1 mAb or control rat IgG2b. Then, serum GOT and GPT levels were examined. n = 3 mice in each group. *P < 0.05, **P < 0.01, ***P < 0.001. *n.s.* not significant. Results are presented as Means ± standard error of the mean (SEM) (C to E). Data derived from at least N = 2 independent experiments.

We have established the experimental mice hepatic IRI model that induce massive cell death in the liver (Fig. 1A,B). Infiltrated population of T lymphocytes, neutrophils, monocytes were quantified at several time points after reperfusion. The results showed that neutrophils are substantially major rather than other leukocytes (Fig. 1C). To examine the role of neutrophil for hepatic IRI, we treated the mice with anti-Gr-1 mAb to deplete neutrophils at 6 and 4 days prior to liver surgery^15^. Flow cytometric analysis confirmed that anti-Gr-1 mAb treatment effectively depleted neutrophils, but not monocytes in peripheral blood mononuclear cells (PBMCs) (Fig. 1D). In this neutrophil-depletion model, the results provided efficient protection with significant reduction in both serum glutamic oxaloacetic transaminase (GOT) and glutamic pyruvic transaminase (GPT) levels (Fig. 1E). These results confirmed that neutrophils play a critical role for hepatic IRI.

### Transient upregulation of CD321 upon hepatic IRI

**Figure 2:**
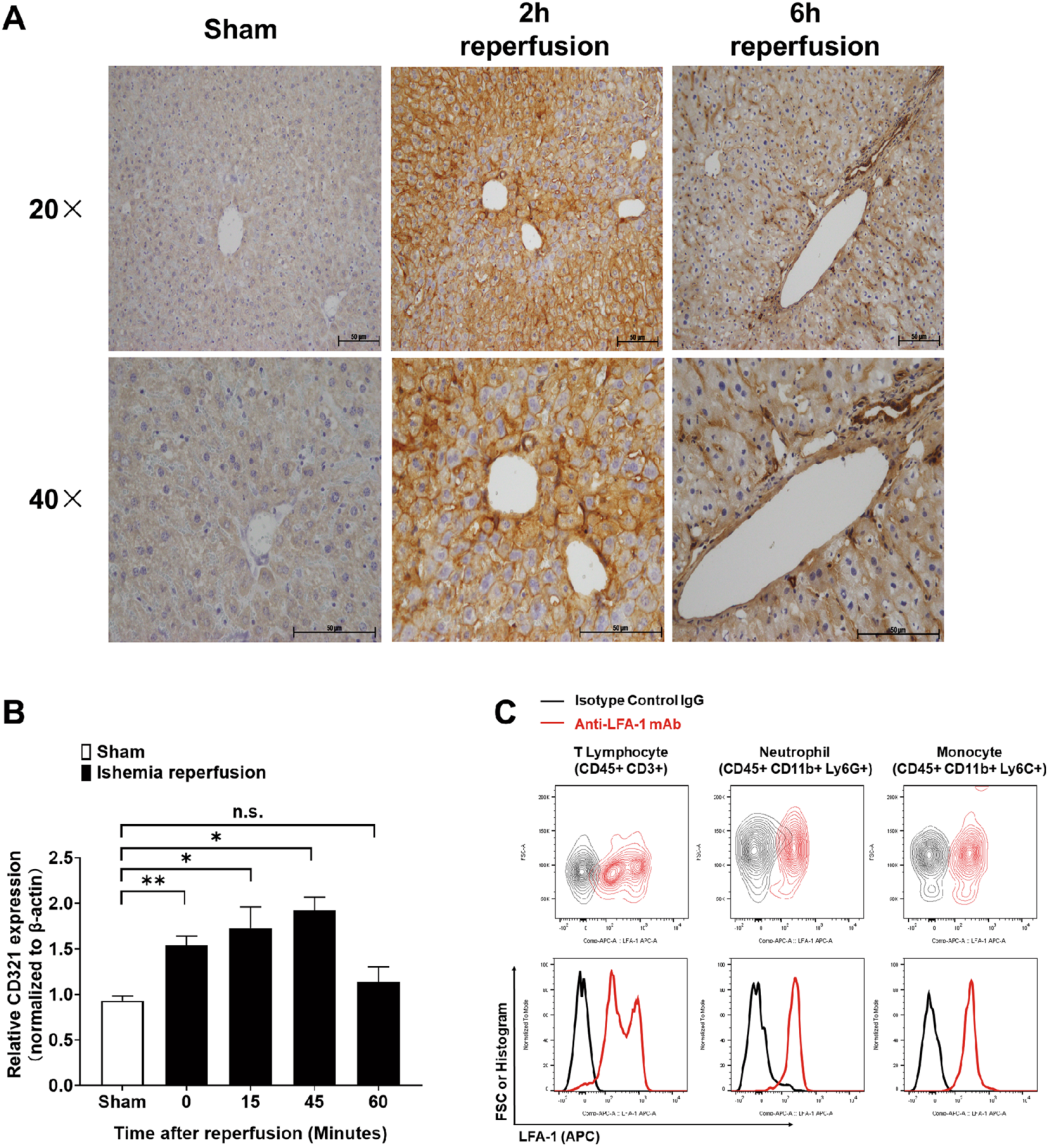
Transient CD321 expression in liver underwent IRI. **(A)** Representative immunohistochemical staining images for CD321 (dark brown staining) in damaged liver. Livers were extracted from the sham-operated mice and the IRI mice at 2 and 6 h after reperfusion. Magnification, × 20 and × 40. Image of the liver of the sham-operated mice were not distinguishable between 2 and 6 h after the sham-operation, thus the image of the liver at 6 h after the sham-operation is demonstrated. **(B)** Relative CD321 mRNA expression levels in livers. mRNAs were obtained from the ischemic lobes at the indicated time after reperfusion and from the sham-operated liver at 30 min after sham-operation. n = 3 in each group. *P < 0.05, **P < 0.01. n.s., not significant. Results are presented as Means ± SEM. **(C)** Representative expression profile of LFA-1 (ligand of CD321) on indicated leukocytes infiltrating in liver after IRI. n = 3 in each group. Infiltrating leukocytes were prepared from liver at 2 h after reperfusion, then LFA-1 expression of the indicated leukocytes population was examined by flowcytometry as described in methods. Data derived from N = 2 experiments.

Expression of CD321 is characterized in various cell types in tissues including the vasculature under physiological and disease conditions, and CD321 has been reported to play critical roles for neutrophil infiltration into the ischemic region^13,16^. Immunohistochemical staining using 90G4 antibody demonstrated increased CD321 expression on hepatic sinusoid endothelium 2 and 6 h after reperfusion in the hepatic IRI model. There was no staining in the sham-operated mice by immunohistochemical analysis (Fig. 2A). This finding was further confirmed by the quantitative polymerase chain reaction (qPCR) analysis, demonstrating that CD321 expression was immediately upregulated in response to ischemia (0 min) with sustained expression during reperfusion phase until 60 min, when compared with the sham-operated mice (Fig. 2B).

Moreover, we examined the expression level of lymphocyte function-associated antigen 1 (LFA-1 known as CD11a/CD18 or αLβ2) known as a primary ligand of vascular CD321 for diapedesis of leukocytes into inflammatory sites^10,11,17^. In damaged liver at 2 h after reperfusion, the results clearly showed that LFA-1 was expressed on T lymphocytes, neutrophils and monocytes (Fig. 2C). These results suggest that CD321 might play a role to regulate the infiltration of inflammatory cells in an early-phase of hepatic IRI.

### Anti-CD321 mAb treatment inhibited leukocyte infiltration into the liver after IRI

**Figure 3:**
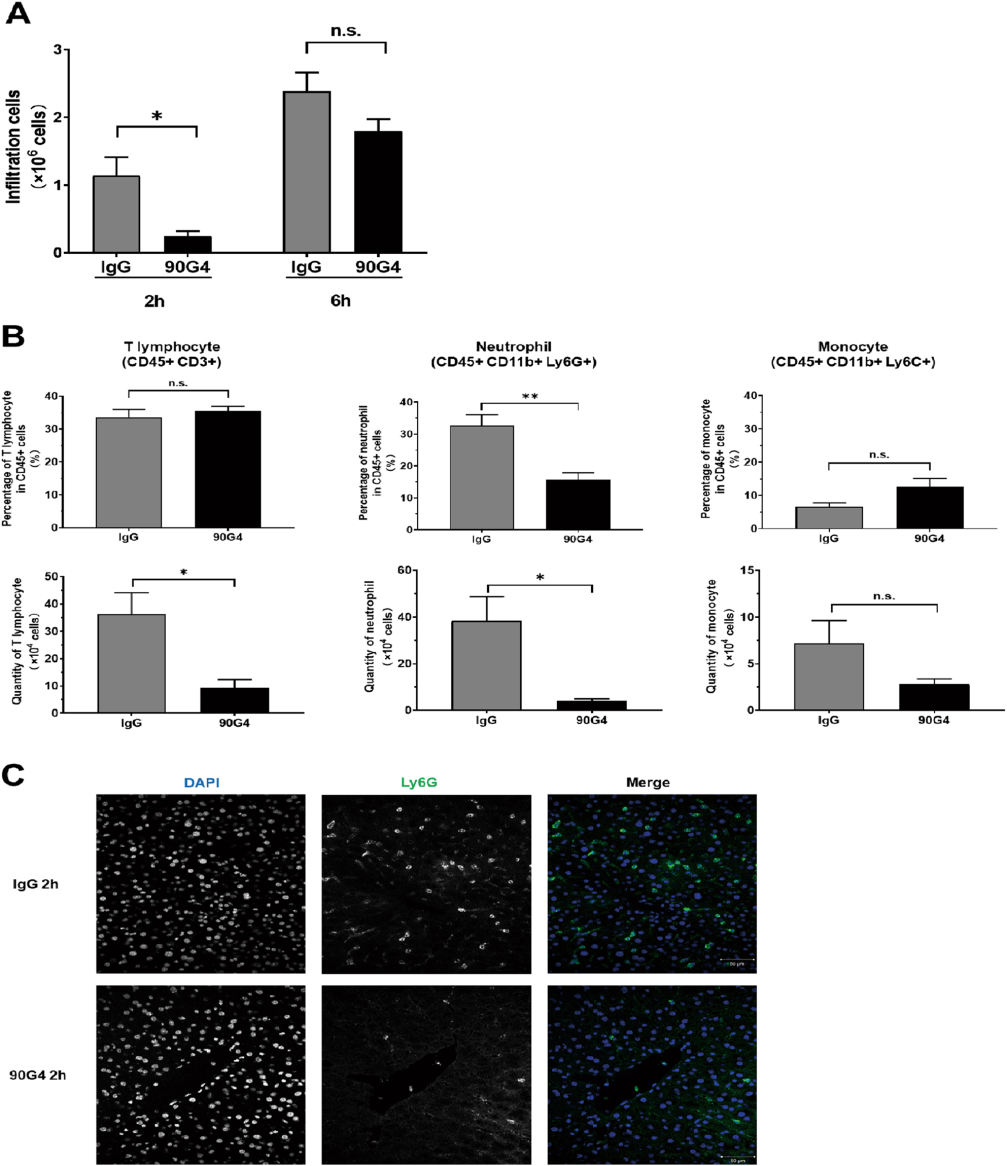
Anti-CD321 mAb (90G4) inhibited leukocyte infiltration in liver after IRI. **(A)** Quantity of nonparenchymal cells in liver suffered IRI. Nonparenchymal cells were prepared from ischemic liver of the mice treated with indicated mAb at 2 h and 6 h after reperfusion. **(B)** Percentage and quantity of intrahepatic infiltrating T lymphocytes, neutrophils and monocytes from the mice treated with indicated mAb at 2 h after reperfusion. (**C**) Representative images of immunocytochemical analysis of Ly6G positive cells, neutrophil, in ischemic liver at 2 h after reperfusion. Cryosections were stained with DAPI (blue) and anti-Ly6G antibody (green). Scale bar, 50 μm. Magnification, × 40. n = 5 in each group. *P < 0.05, **P < 0.01. *n.s.* not significant. Results are presented as Means ± SEM.

Given the evidence of CD321 upregulation in ischemic liver, we hypothesized that LFA-1 positive lymphocytes infiltrate via LFA-1/CD321 molecular interaction. To inhibit trans-endothelial migration and subsequent liver IRI, we evaluated the therapeutic efficacy of anti-CD321 mAb (90G4). The antibody was administrated through the portal vein after the removal of the micro-clip for reperfusion, subsequently, the infiltrating non-parenchymal cells in the livers were quantified. The treatment with 90G4 mAb reduced the number of non-parenchymal cells significantly at 2 h, but not 6 h after reperfusion compared with IgG-treated mice (Fig. 3A). At 2 h after reperfusion, we found that the infiltration of CD45^+^ cell population, including neutrophils, T cells and monocytes, appeared to be decreased in number by 90G4 antibody administration. When this result was expressed as percentage of CD45^+^ cells, the infiltration of neutrophil, but not T cell or monocyte, was significantly decreased (Fig. 3B). The liver sections were subjected for immunohistochemical staining with anti-Ly6G antibody which specifically detect neutrophil, resulting in confirmation of dramatic reduction of infiltrated neutrophil in 90G4 antibody treated group (Fig. 3C). At 6 h after reperfusion, 90G4 treatment did not alter the percentage or quantity of T cells and neutrophils in the ischemic lobes, nevertheless, mice treated with 90G4 mAb had a lower percentage and number of monocytes with statistically significance (Fig. S1). Thus, 90G4 mAb inhibited the interaction between neutrophilic LFA-1 and vascular CD321 at an early phase after reperfusion, implying for the therapeutic potency of CD321 blockade.

### Anti-CD321 mAb treatment attenuated hepatic IRI

**Figure 4:**
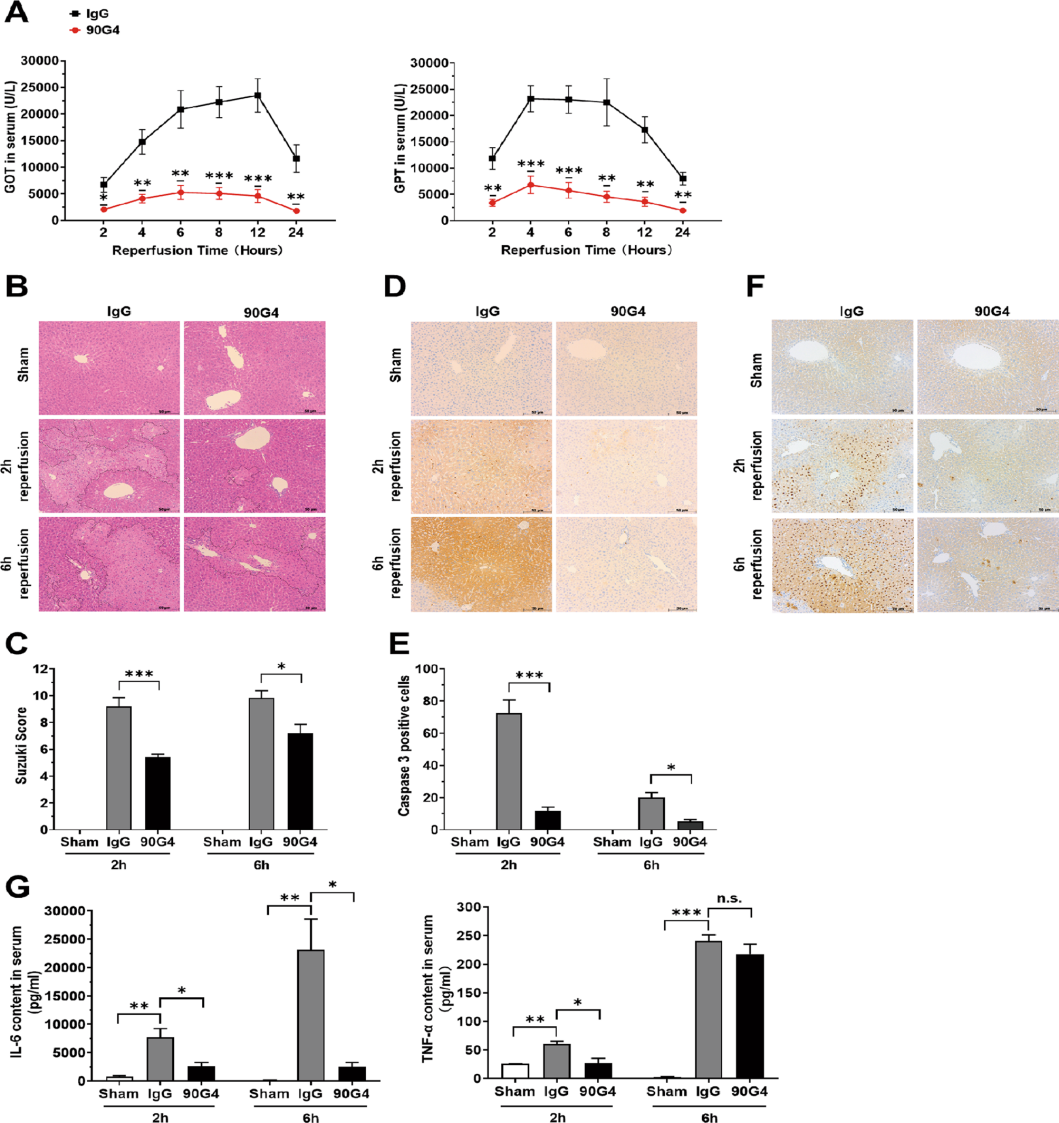
Anti-CD321 mAb (90G4) treatment inhibits hepatic IRI. **(A)** Serum GOT and GPT levels in 90G4-tretaed hepatic IRI mice. Sera were collected at the indicated time after the reperfusion from the hepatic IRI mice treated with 90G4 or rat IgG, then serum GOT and GPT levels were examined (n = 5–7 in each group). **(B)** Representative images of hematoxylin and eosin staining of damaged livers from indicated antibody-treated mice at indicated times after reperfusion. Necrotic areas are surrounded by dashed lines. **(C)** Histologic injury score of hematoxylin and eosin staining sections analyzed by Suzuki criteria (n = 3 mice in sham group and n = 5–6 mice in IRI group). **(D)** Representative images of Caspase-3 staining of livers at the indicated time after reperfusion of the indicated antibody-treated mice. **(E)** Caspase-3 positive cells (dark brown spots in Figure **D**) were counted and analyzed using Image J software (n = 3 mice in sham group and n = 5–6 mice in IRI group). **(F)** Representative images of TUNEL staining of ischemic livers at the indicated time after the reperfusion of the indicated antibody-treated mice (n = 3 in each group). **(G)** Serum IL-6 and TNF-*α* levels. Sear was collected from the sham-operated mice and IRI mice treated with indicated mAb at 2 and 6 h after the reperfusion. The amount of IL-6 and TNF-*α* was measured by ELISA (n = 3 mice in sham group and 5–6 mice in IRI group). *P < 0.05, **P < 0.01, ***P < 0.001. Results are presented as Mean ± SEM (**C**,**E**,**G**). Magnification, × 20 (**B**,**D**,**F**). N = 2–3 independent experiments.

We revealed that neutrophil depletion by Gr-1 mAb treatment is necessary for the protection of hepatic IRI. Because vascular CD321 was rapidly upregulated in liver upon ischemia, blocking LFA-1/CD321 is a potent target of therapeutics for hepatic protection. In fact, inhibition of CD321 by 90G4 mAb suspended neutrophil infiltration at an early phase after hepatic IRI. We next examined whether 90G4 mAb is sufficient for the protection of hepatic IRI. The result showed that 90G4 mAb treatment significantly reduced serum GOT and GPT levels from 2 to 24 h after reperfusion compared with the IgG-treated mice (Fig. 4A). Suzuki score^18^ demonstrated that necrotic regions were significantly reduced in the liver of 90G4-treated mice (Fig. 4B,C). Consistently, the protected liver showed the reduced number of Caspase-3 positive cells as well as terminal deoxynucleotidyl transferase–mediated deoxyuridine triphosphate nick end labeling (TUNEL) positive cells (Fig. 4D–F).

As the detail mechanism of hepatic protection by 90G4 mAb treatment, we examined serum level of interleukin (IL) -6 or tumor necrosis factor (TNF) -α, inflammatory cytokines reported to play a key role to activate infiltrating leukocytes in the inflamed liver^19,20^. 90G4 mAb treatment significantly lowered serum IL-6 level at 2 and 6 h after reperfusion and serum TNF-α level at 2 h after reperfusion respectively (Fig. 4G). In summary, these results presented that 90G4 administration ameliorated hepatic IRI by inhibiting the infiltration of neutrophil, which is a major producer of inflammatory cytokines.

## Discussion

Although IRI is induced by both oxidative stress after blood resupply, irreversible damage of hepatocyte is caused mainly by inflammatory leukocytes migrating into the liver^21^. However, current therapeutic approaches are limited and unsatisfactory. Here, we demonstrated that infiltrating neutrophils played an essential role in triggering hepatic IRI. CD321 immunotherapy using 90G4 antibody provided dramatic suppression of hepatic IRI via decreased number of leukocytes, especially in the early phase after reperfusion.

It has been shown that hepatic IRI composed of two distinct phases of innate immune responses^22^. The initial phase (< 2 h after reperfusion) involves Kupffer cell activation to produce free radicals, cytokines, and chemokines^20^. In the late phase, the produced cytokines activate infiltrated leukocytes, leading to the irreversible damage of hepatocytes. Kupffer cells are characterized as the main source of primary inflammatory mediator, such as TNF-α^23,24^, in hepatic IRI. As an alternative role of TNF-α, it has been shown that TNF-α stimulates to induce CD321 redistribution on endothelial cells, assisting neutrophil transmigration to the inflamed tissue^25^. In the late phase, neutrophils also produce TNF-α^26^, but a central role of TNF-α is to induce simultaneous inflammation like IL-6 production mediated by monocytes and endothelial cells^27^, leading to exaggerate the liver injury further^28^. Thus, 90G4 mAb mediated-inhibition of neutrophil infiltration at early phase would be extremely beneficial for subsequent suppression of TNF-α production in the late phase to cause hepatic damage.

It has been considered that leukocytes infiltration are regulated in organ-specific and cell-type specific mechanism^29^, but this is because the multi-steps in diapedesis are under the strict control by multiple surface molecules. For example, CD321 plays an essential role for transmigration at the final step of diapedesis into inflammatory tissues underwent heart IRI model^12^. Similarly, our presented results in hepatic IRI model demonstrated that CD321 blockade inhibited neutrophil infiltration and hepatic injury. 90G4 mAb treatment suspended all the injury markers: necrosis and apoptosis of hepatocytes, secretion of GOT and GPT, upregulation of TNF-α and IL-6.

Previous experiments utilizing CD321-deficient mice demonstrated that apoptotic hepatocytes were increased despite the suppression of neutrophil infiltration upon hepatic IRI. In this case, serum GOT and GPT levels were not changed^13^. We firstly need to understand that the inhibition of CD321 function by either knockout animal or blocking antibody provide a different situation in terms of mechanism. Notably, presented results demonstrated that inhibition of neutrophil infiltration at an early phase sufficiently reduced hepatic IRI. Thus, transient inhibition of CD321 by 90G4 mAb might have advantage to inhibit hepatic injury in our IRI model. Since 90G4 mAb reacts in-situ in the context of pathophysiological processes unlike constitutive knockout, it reminds that CD321 is the bona fide therapeutic target in hepatic IRI. Further studies are required to reveal the mechanisms of monocyte infiltration upon hepatic IRI and the role of infiltrating monocytes for hepatic IRI. Besides, it also has to be considered the underlying mechanism by junctional adhesion molecule (JAM) family members for regulating tight junction. Given the evidence that JAM-C plays a crucial role on tissue repairing, the interplay of CD321 with other JAM family member could be important especially at the resolution phase (several days after reperfusion)^30,31^.

CD321 is redistributed and internalized in endothelial cells upon hypoxic stimulation or inflammatory stimulation^32,33^. As a therapeutic target, dual roles of CD321 for tight junction formation and lymphocyte diapedesis upon stimuli provide potent characters in inflammation-dependent accessibility of immunotherapeutics. In fact, CD321 is a proven therapeutic target of peritonitis, skin inflammation, meningitis, and heart IRI^11,34–36^. In summary, our findings propose that disrupting human LFA-1/CD321 interaction is the useful and attractive strategy to inhibit trans-endothelial migration of neutrophils for protecting hepatic IRI in clinic.

## Methods

### Study design

The aim of this study is to investigate the effect of intraportal administration of anti-CD321 monoclonal antibody (90G4) with regard to liver IRI in a murine model. Group size was determined by power analysis using the effect size from pilot experiments. All mice in this study were randomized before treatment. To ascertain the mechanism of acute phase in hepatic IRI, we set end points at 24 h after reperfusion in vivo study. By taking careful consideration of abnormal anatomy of mice, mice that failed to establish at least 70% of damage after liver IR due to anatomically abnormal structure were excluded from the analysis (Fig. S2). The researchers were blinded to conduct of the experiment and assessment of outcomes.

### Reagents

Rat anti-mouse CD321 mAb, clone 90G4^32^, and anti-Gr-1 mAb, clone RB6-8C5^37^, were prepared and purified in our laboratory as previously described. As a control antibody, rat IgG was purchased from Southern Biotech (Birmingham, AL, USA). All antibodies used in this study was dialyzed in 0.9% NaCl prior to injection.

### Animal liver IRI model

Four- to six-week-old wildtype male C57BL/6J mice were purchased from Charles River Japan Inc. (Yokohama, Japan) and maintained in the specific pathogen-free facility at Juntendo University. Eight- to ten-week-old mice were subjected to establish a warm 70% liver IRI model as described in the literature^3,38–42^ (Fig. 1A). Briefly, mice were anesthetized through intraperitoneal injection with pentobarbital sodium. A micro-clamp was placed at porta hepatis right above the branching to the right lateral lobe to interrupt the blood flow for 1 h. Mice body temperature was maintained at 37 °C on a heating plate. Either 90G4 antibody or rat IgG (300 μg per mouse) was injected through the portal vein promptly after removing the micro-clamp. All the same procedure but flow occlusion was performed in the mice of sham group. All protocol was approved by Medicine Animal Ethics Committee of Juntendo University (Approved number 2020131). All animal experiments were performed in compliance with the applicable national laws and regulations, the institutional guidelines and ARRIVE guidelines on animal experimentation. All the experimental protocols involving animals are reviewed by the institutional animal care and use committee.

### Histopathology and immunohistochemical analysis

Ischemic liver lobes were harvested and snap frozen in liquid nitrogen at 2 and 6 h after reperfusion. Liver sections with 6 μm of thickness were fixed with 4% paraformaldehyde in phosphate buffer solution, in the following treatment with 0.3% hydrogen peroxidase to inhibit endogenous peroxidase activity. Sections were incubated with rat anti-mouse 90G4 antibody (2 μg per section) as primary antibody overnight, and biotin-streptavidin donkey anti-rat IgG (H + L) (1 μg per section) as secondary antibody for 2 h. Then, samples were incubated with pre-formed streptavidin/biotin complex (ABC kit, Nacalai Tesque, Kyoto, Japan), and subsequent colorimetric development with diaminobenzidine substrates as the manual instructed (DAB kit, Nacalai Tesque, Kyoto, Japan).

Hematoxylin and eosin (H&E) staining was performed as described previously^43^. Stained sections were analyzed in a double-blinded manner. A Suzuki score was calculated based on evaluation of congestion, vacuolization, and necrosis^18^. In the immunohistochemical evaluation of apoptotic cell death, tissue sections were stained with rabbit anti-cleaved caspase-3 (Asp175) (Cell Signaling Technology, Denvers, MA, USA) as described previously^44^. TUNEL staining was employed for paraffin-embedded liver sections as described previously^45^. Cryosections of the ischemic liver at 2 h reperfusion were stained with anti-mouse Ly6G (Clone 1A8) (Biolegend, San Diego CA, USA), which was followed by staining with Alexa Fluor 488-conjugated anti-rat IgG (H + L) (Invitrogen). Sections were counterstained with DAPI (Invitrogen)^46^. Images were captured by AxioVision software of Zeiss Axio Lab.A1 microscope with attached AxioCam ERc 5S. Fluorescent images were obtained and analyzed using a FV300 confocal laser microscopy (Fluoview laser scanning biological microscope JX70 system; Olympus, Albertslund, Denmark).

### Analysis of CD321 transcript by qPCR

After one hour of blood flow occlusion, the treated livers were harvested at each time point (15, 45 and 60 min) after reperfusion, and immediately fixed in TRI Reagent (TR118, Cosmo Bio, Tokyo, Japan). Total RNA was isolated from the tissues by RNeasy Mini kit (Qiagen, Tokyo, Japan), then total RNA (0.1 μg per sample) was subjected to reverse transcription to cDNA in 8 μl of reaction volume using an qPCR kit (Toyobo, Osaka, Japan) according to the instruction manual. Real-time qPCR was performed for CD321 gene (Mm00554113_m1) and β-actin (Mm00607939_s1) with a TaqMan Universal PCR Master Mix on the StepOnePlus System (Applied Biosystems of Thermo Fisher Scientific, Waltham, Mass, USA). Quantitative level of CD321 mRNA transcript was normalized to mouse β-actin mRNA expression by a comparative C_T_ method.

### Quantification of injury level by measurement of serum liver enzymes

Peripheral blood (P.B.) samples were collected via tail at 2, 4, 6, 8, 12 and 24 h after reperfusion, and centrifuged at 1300×g for 20 min. Serum samples were retrieved and stored in a − 80 °C deep freezer until measurement. Levels of GOT and GPT were measured by using a dri-chem slide kit on a Multi-purpose automatic dry-chemistry analyzer (Fujifilm, Tokyo, Japan).

### Flow cytometry analysis

Non-parenchymal cells were isolated from liver as previously described^47^. Briefly, after perfusion with 30 ml of phosphate-buffered saline (PBS), liver was grinded on a stainless-steel mesh and suspended in PBS containing 3% fetal bovine serum. After single centrifugation at 820×g for 10 min, pellet was suspended in the 30% Percoll for further separation with centrifugation at 660×g for 18 min at room temperature without brakes. The pellet was resuspended in red blood cell-lysis buffer for hemolysis, then washed once in PBS containing 3% fetal bovine serum. For staining, cells were incubated with viability dye and fluorochrome-conjugated antibodies: Zombie dye (Zombie Violet Fixable Viability Kit), CD45.2 (PerCP; clone 104), CD3 (PE/Cy7 or PE; clone 17A2), CD11b (Violet 421 or PE; clone M1/70), Ly6G (APC or APC/Cy7; clone 1A8), Ly6C (PE or FITC; clone HK1.4), F4/80 (FITC; clone BM8), and LFA-1 (APC; clone H155-78). All reagents were purchased from BioLegend (Biolegend, San Diego, CA, USA). Flow cytometry analysis was performed by BD FACSVerse, and data were analyzed with FlowJo software (BD Biosciences, San Jose, CA, USA). For identifying specific cell types, gating strategy was subjected to define T lymphocyte (CD45^+^CD3^+^), neutrophil (CD45^+^CD11b^+^Ly6G^+^), and monocyte (CD45^+^CD11b^+^Ly6C^+^) respectively.

### Enzyme linked immunosorbent assay (ELISA)

Serum samples were collected at 2 and 6 h after liver IRI. The concentration of IL-6 or TNF-α in serum was detected separately by corresponding Quantikine ELISA kit (R&D Systems, Minneapolis, MN, USA) following the manufacturer’s instructions.

### Neutrophil depletion

Circulating neutrophil was depleted via intraperitoneal injections of anti Gr-1 mAb (100 μg per mouse) on the day 0 and 2, which are corresponding to 6 and 4 days prior to induce ischemia^15^. The quantity of neutrophil and monocyte in P.B. were analyzed by flow cytometry to confirm the accomplishment of depletion on the day 6 after the first injection.

### Statistical analysis

Obtained data were visualized by Prism software in graph with means ± SEM. Statistical differences between two groups were assessed by an unpaired Student’s *t* test. *P* value of less than 0.05 was regarded as statistically significant.

## Supplementary Information

Supplementary Information.
