# Supplementary material for: Anti-CD321 antibody immunotherapy protects liver against ischemia and reperfusion-induced injury
Source: Sci Rep. 2021 Mar 18;11:6312. doi: 10.1038/s41598-021-85001-2 (PMC7973783; doi:10.1038/s41598-021-85001-2)
Supplement: Supplementary file 1 — Supplementary Information. [file 41598_2021_85001_MOESM1_ESM.pdf]

Anti-CD321 antibody immunotherapy protects liver against ischemia and reperfusion-induced injury

Authors: Enzhi Yin[1], Takeshi Fukuhara[2]*, Kazuyoshi Takeda[3,4], Yuko Kojima[5], Kyoko Fukuhara[6], Kenichi Ikejima[6], Hisashi Bashuda[1], Jiro Kitaura[1], Hideo Yagita[7], Ko Okumura[1,4], Koichiro Uchida[8]*

Affiliations:

[1]Atopy Research Center, Juntendo University Graduate School of Medicine, Tokyo, Japan.

[2]Department of Neurology, Juntendo University School of Medicine, Tokyo, Japan.

[3]Laboratory of Cell Biology, Research Support Center, Juntendo University Graduate School of Medicine, Tokyo, Japan.

[4]Department of Biofunctional Microbiota, Juntendo University Graduate School of Medicine, Tokyo, Japan.

[5]Laboratory of Morphology and Image Analysis, Research Support Center, Juntendo University Graduate School of Medicine, Tokyo, Japan.

[6]Department of Gastroenterology, Juntendo University Graduate School of Medicine, Tokyo, Japan.

[7]Department of Immunology, Juntendo University School of Medicine, Tokyo, Japan.

[8]Juntendo Advanced Research Institute for Health Science, Juntendo University School of Medicine, Tokyo, Japan.

*Corresponding author. Email: k-uchida@juntendo.ac.jp (K.U.); noantibody-noscience@umin.ac.jp (T.F.)

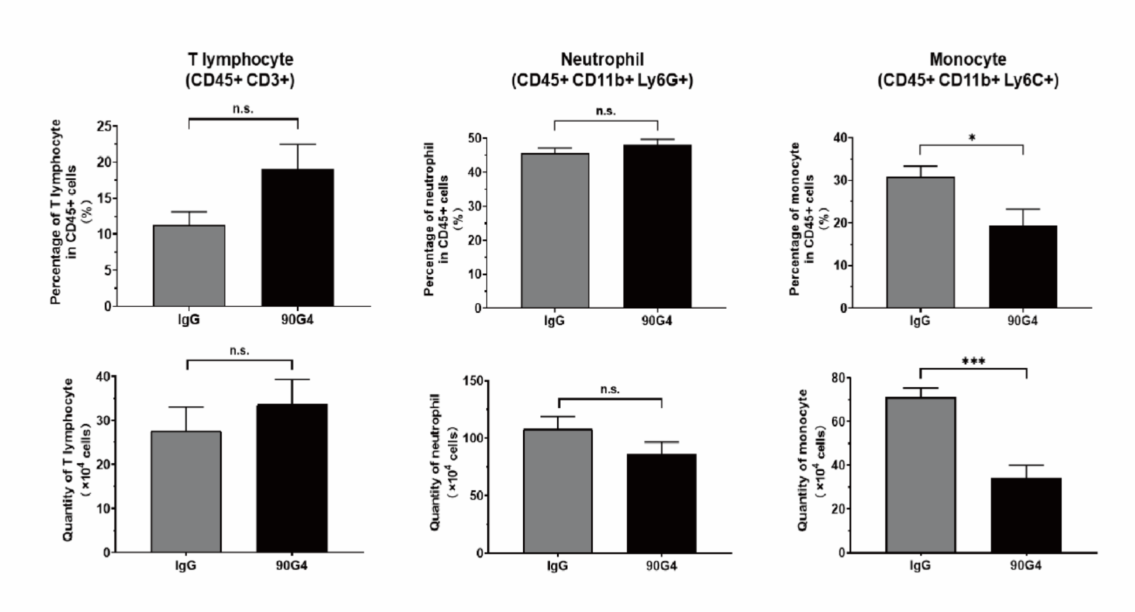

Figure S1. Percentage and quantity of intrahepatic infiltrating T lymphocytes, neutrophils and monocytes from the mice treated with indicated mAb at 6 hours after reperfusion. *n* =4-6 in each group. * $P < 0.05$, *** $P < 0.001$. n.s., not significant. Results are presented as Means ± SEM.

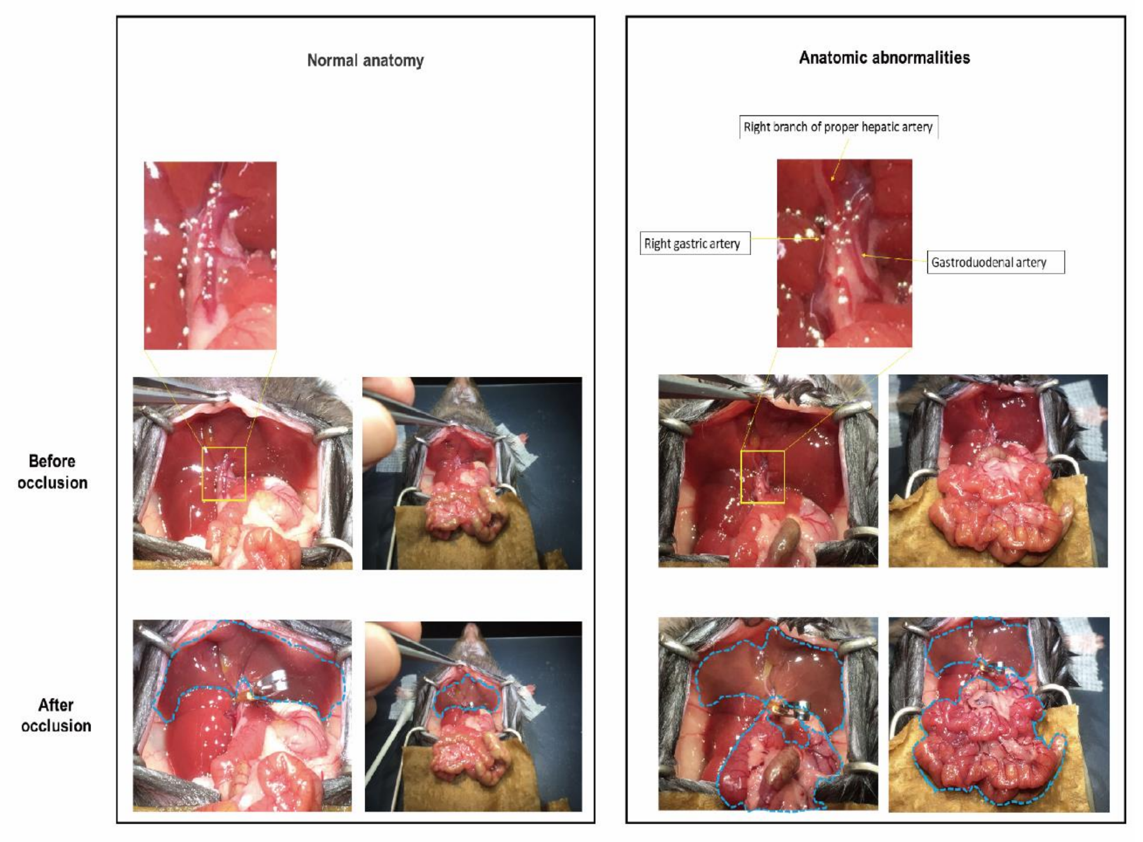

Figure S2. The photograph of normal anatomy and anatomic abnormalities in establishing the murine IRI model. Ischemic areas are surrounded by blue dashed line.

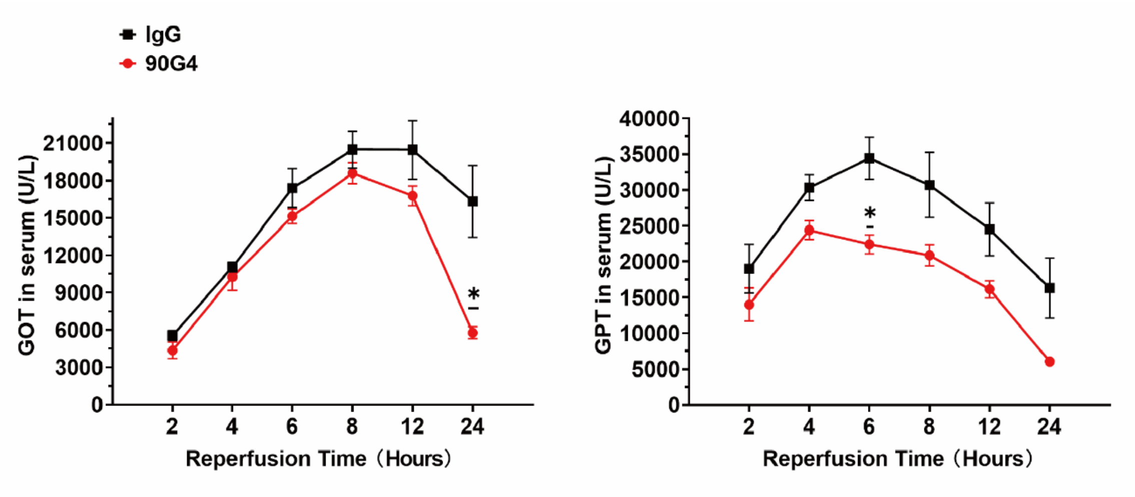

Figure S3. Sera were collected at the indicated time after reperfusion from the hepatic 90 min ischemia mice treated with 90G4 mAb or control IgG. Then, serum GOT and GPT levels were examined. $n = 4$ mice in each group. * $P < 0.05$, Results are presented as Means ± standard error of the mean (SEM)
